# Gefitinib successfully administered in a lung cancer patient with leptomeningeal carcinomatosis after erlotinib-induced pneumatosis intestinalis

**DOI:** 10.1186/s12885-018-4743-5

**Published:** 2018-08-16

**Authors:** Hironori Uruga, Shuhei Moriguchi, Yui Takahashi, Kazumasa Ogawa, Kyoko Murase, Sayaka Mochizuki, Shigeo Hanada, Hisashi Takaya, Atsushi Miyamoto, Nasa Morokawa, Kazuma Kishi

**Affiliations:** 1 Department of Respiratory Medicine, Respiratory Center, Toranomon Hospital, 2-2-2 Toranomon, Minato-ku, Tokyo, 105-8470 Japan; 2 Okinaka Memorial Institute for Medical Research, Tokyo, Japan

**Keywords:** Pneumatosis intestinalis, Epidermal growth factor receptor tyrosine kinase inhibitor, Leptomeningeal carcinomatosis, Non-small-cell lung cancer

## Abstract

**Background:**

Pneumatosis intestinalis (PI) is a rare complication of chemotherapy, characterized by multiple gas accumulations within the bowel wall.

**Case presentation:**

A 71-year-old woman with epidermal growth factor receptor (EGFR) mutation-positive lung adenocarcinoma was admitted to our hospital because of reduced consciousness. She was diagnosed as having leptomeningeal carcinomatosis (LM) using lumbar puncture. Because she could not swallow a tablet, erlotinib was administered via a feeding tube. Her state of consciousness gradually improved, but she experienced diarrhea several times a day. After 3 weeks of erlotinib therapy, PI occurred. Erlotinib was discontinued and PI was resolved after treatment with conservative therapies. Erlotinib was re-administrated and PI occurred again. After improvement of erlotinib-induced PI, gefitinib was administered by a feeding tube and the patient did not experience PI or diarrhea. The patient survived 8 months from the diagnosis of LM.

**Conclusion:**

PI is one of the side effects of erlotinib, and consecutive therapies are useful for the treatment of PI. In this patient, gefitinib was successfully administered after erlotinib-induced PI.

## Background

Pneumatosis intestinalis (PI) is a rare complication of chemotherapy including cytotoxic and molecular-targeted agents [1], and is characterized by multiple gas accumulations within the bowel wall. PI mainly occurs when gas enters the submucosal and subserosal tissue of the bowel wall with infiltration of giant cells [2]. The gas is thought to come from the intraluminal gastrointestinal system, bacterial production, or a pulmonary source [3]. Besides chemotherapy, PI is associated with various medical conditions, such as collagen-vascular diseases, lupus enteritis, infectious colitis, asthma, acquired immune deficiency syndrome, cystic fibrosis, hematopoietic stem cell transplantation, trauma, and steroid therapy [2–4]. Compared with cytotoxic agents, PI due to molecular-targeted agents is even rarer [1, 5–12], and only several cases have been reported in association with epidermal growth factor receptor (EGFR) tyrosine kinase inhibitors (TKIs) [1, 13–17]. Here, we describe a patient with leptomeningeal carcinomatosis (LM) caused by EGFR-mutated lung adenocarcinoma who was treated with gefitinib via a feeding tube after erlotinib-induced PI.

## Case presentation

**Fig. 1:**
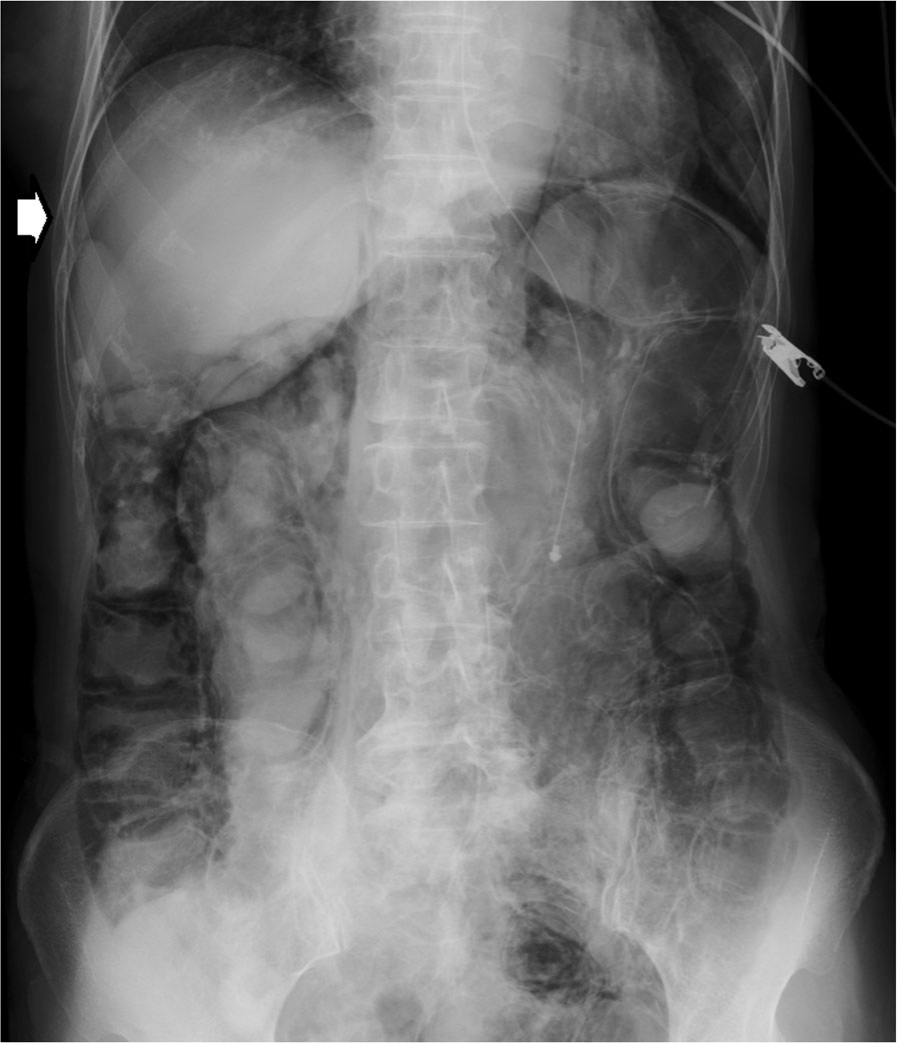
An abdominal radiograph showed a dilated colon and the presence of intraluminal air along the wall of the colon with free air (arrow)

**Fig. 2:**
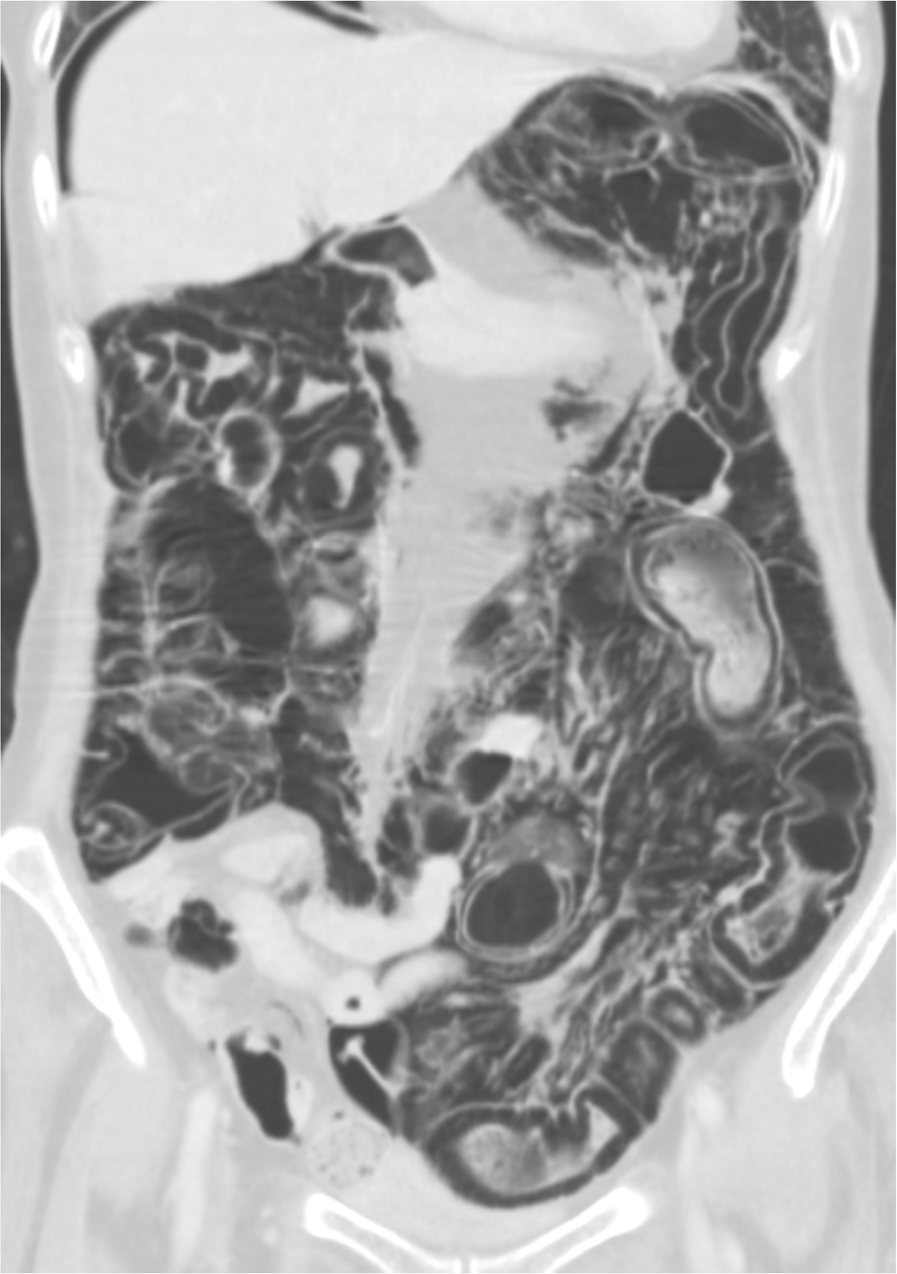
A CT scan of the abdomen demonstrated a thickening of the colon with free air, pneumoretroperitoneum, and pneumomediastinum

A 71-year-old Japanese woman was admitted to Toranomon hospital because of reduced consciousness. She had a history of a mastectomy for right breast cancer. Eight months before admission, she came to our hospital because of partial paralysis of her right hand. A chest computed tomography (CT) scan showed a mass in the left upper lobe, as well as mediastinal and left hilar lymphadenopathy. Brain magnetic resonance imaging (MRI) showed multiple brain metastases. The patient was diagnosed with EGFR mutation-positive adenocarcinoma using transbronchial lung biopsy. She received 4 cycles of carboplatin and paclitaxel chemotherapy, and gamma knife treatment followed by whole brain radiation for brain metastases. Although chemotherapy resulted in a partial response, her state of consciousness rapidly worsened in the 2 weeks before admission. Her level of consciousness on admission was a Glasgow Come Scale (GCS) score of 8. She could not speak or move her extremities. A lumbar puncture was performed and cytological examination of her cerebrospinal fluid revealed adenocarcinoma cells. EGFR mutation analysis of cerebrospinal fluid was positive for L858R, but negative for exon 20 T790 M. Erlotinib at 150 mg/day was dissolved in 15 mL of water, and was administered via a feeding tube because she could not swallow a tablet. At the same time, 8 mg/day dexamethasone and glycerin administration were started via drip infusion. The patient’s state of consciousness gradually improved to a GCS score of 13, and serum levels of CA19–9 decreased from 3031 U/mL to 292 U/mL. As a side effect of erlotinib, diarrhea of the Common Terminology Criteria for Adverse Events (CTCAE) version 4.0 grade 2 developed. Three weeks after receiving erlotinib and steroids, the patient started to vomit and suffered from grade 3 diarrhea. At that time, her body temperature, blood pressure, and pulse rate were 36.8 °C, 105/55 mmHg and 92 b.p.m., respectively. There was no abdominal tenderness on her physical examinations. The laboratory test results showed a normal leukocyte count (4700/mL), and a slightly increased C-reactive protein level (2.4 mg/dL). Arterial blood gas analysis showed alkalemia (pH 7.50). An abdominal radiograph showed a dilated colon and the presence of intraluminal air along the wall of the colon (Fig. 1). A CT scan of the abdomen demonstrated pneumatosis; there was a thickening of the colon with free air, pneumoretroperitoneum, and pneumomediastinum (Fig. 2). There were no signs of bowel ischemia or portal venous air. Erlotinib and steroids were stopped, and the patient was managed conservatively with a combination of antibiotics and inhalation of oxygen. Two weeks later, the CT findings of PI were improved. Erlotinib was re-administered without steroids, and PI relapsed 4 weeks later with diarrhea. After improving PI by discontinuation of erlotinib and conservative therapies, gefitinib was administered safely without PI and diarrhea for 3 month. At that time, the EGFR-TKIs afatinib and osimertinib were available in the Japanese market. The number of cells in the cerebral fluid decreased from 132/μL to 37/μL with erlotinib and gefitinib, although the cytology was still positive for adenocarcinoma. The patient died of worsening brain metastases 8 months after the diagnosis of LM.

## Discussion

We describe a patient with LM from EGFR-mutated lung adenocarcinoma who was treated with erlotinib followed by gefitinib through a feeding tube for 8 months. As adverse events of erlotinib, PI and grade 3 diarrhea developed, both of which were resolved after treatment with conservative therapies. Subsequently, gefitinib could be administered without either PI or diarrhea.

The precise mechanism by which gefitinib was administered safely instead of erlotinib in this patient is unclear. In comparison of side effects between erlotinib and gefitinib, hepatotoxicity was more common in gefitinib, but rash was more common in erlotinib; the incidence of diarrhea and interstitial lung disease was almost the same [18]. However, there have been several reports of successful treatments with erlotinib after gefitinib-induced interstitial lung disease [19–22]. One of these reports suggested the hypothesis that minor differences in the chemical structure between two drugs binding to the common quinazoline and anilino rings might influence the results [20].

PI can be caused by various agents, such as cyclophosphamide, methotrexate, vincristine, doxorubicin, daunorubicin, cytarabine, fluorouracil, paclitaxel, docetaxel, etoposide, irinotecan, 5 fluorouracil (5-FU), and cisplatin [12, 14]. PI secondary to molecular-targeted agents is very rare [1, 5–14]. Most patients received molecular-targeted therapy alone, and were treated conservatively for PI [1]. Regarding EGFR-TKIs, PI was reported following gefitinib and erlotinib treatment in several case reports [1, 13–17] (Table 1). These patients presented with gastrointestinal symptoms, such as diarrhea or constipation, and PI was improved by conservative therapies and discontinuation of TKIs.

**Table 1:** Previous case reports of pneumatosis intestinalis following gefitinib and erlotinib treatment

Year	Author	Age	Sex	EGFR-TKIs	CT findings	Treatment
2012	Shinagare, et al. 1)	N.D.	N.D.	Erlotinib	N.D.	N.D.
2012	Iwasaku, et al. 13)	82	F	Gefitinib	Intramural air within the intestinal wall	Conservative therapy
2012	Lee, et al. 14)	66	F	Gefitinib	Intramural air within the intestinal wall, portal venous air, Infarction of liver	Conservative therapy
2014	Hughes, et al. 15)	N.D. Three patients	N.D.	Erlotinib and pertuzumab	N.D.	N.D.
2015	Ando, et al. 16)	71	M	Gefitinib	Intramural air within the intestinal wall, ascites	Conservative therapy
2016	Maeda, et al. 17)	80	F	Gefitinib	Intramural air within the intestinal wall, pneumoretroperitoneum,	Conservative therapy
2017	Current case	71	F	Erlotinib	Intramural air within the intestinal wall, pneumoretroperitoneum, pneumomediastinum	Conservative therapy

*EGFR-TKIs* Epidermal growth factor receptor tyrosine kinase inhibitors

*N.D* Not described

The precise mechanism of PI secondary to EGFR-TKIs is unknown. EGFR-TKIs have gastrointestinal toxicities, such as diarrhea, nausea, and vomiting. Gastrointestinal toxicities and the subsequent loss of mucosal integrity of the bowel wall may contribute to PI secondary to EGFR-TKIs. In the present case, we were able to treat the patient with gefitinib and erlotinib-induced PI because she did not experience diarrhea after the switch to gefitinib.

The radiological findings of PI were first reported on abdominal radiograph, and the features were submucosal and subserosal cysts formed in ellipses/circles within the bowel wall [23]. However, nowadays, CT is more useful for the diagnosis of PI [3, 24]. Typical CT findings are submucosal and subserosal air within the bowel wall and free air [25, 26]. Like in our patient, PI is sometimes accompanied by pneumomediastinum, which occurs when retroperitoneal and peritoneal air enters along the para-aortic tissues and exits thorough the esophageal hiatus into the mediastinum [27, 28].

## Conclusions

PI is one of the side effects of EGFR TKIs, and is usually treated with conservative therapies. In this patient, gefitinib was successfully administered after erlotinib-induced PI.

## Abbreviations

5-FU: 5 fluorouracil
CT: Computed tomography
CTCAE: Common Terminology Criteria for Adverse Events
EGFR-TKI: Epidermal growth factor receptor tyrosine kinase inhibitor
GCS: Glasgow Come Scale
LM: Leptomeningeal carcinomatosis
NSCLC: Non-small-cell lung cancer
PI: Pneumatosis intestinalis
